# Rigorous anaesthesia management protocol for patients with intracranial arterial stenosis: a prospective controlled-cohort study

**DOI:** 10.1136/bmjopen-2015-009727

**Published:** 2016-01-19

**Authors:** Azim N Laiwalla, Yinn Cher Ooi, Barbara Van De Wiele, Keren Ziv, Adam Brown, Raymond Liou, Jeffrey L Saver, Nestor R Gonzalez

**Affiliations:** 1 Department of Neurosurgery, David Geffen School of Medicine at the University of California (UCLA), Los Angeles, California, USA; 2 Department of Anesthesiology, David Geffen School of Medicine at the University of California (UCLA), Los Angeles, California, USA; 3 Department of Neurology, David Geffen School of Medicine at the University of California (UCLA), Los Angeles, California, USA; 4 Department of Neurosurgery and Radiology, David Geffen School of Medicine at the University of California (UCLA), Los Angeles, California, USA

**Keywords:** NEUROSURGERY, STROKE MEDICINE

## Abstract

**Objectives:**

Reducing variability is integral in quality management. As part of the ongoing Encephaloduroarteriosynangiosis Revascularisation for Symptomatic Intracranial Arterial Stenosis (ERSIAS) trial, we developed a strict anaesthesia protocol to minimise fluctuations in patient parameters affecting cerebral perfusion. We hypothesise that this protocol reduces the intraoperative variability of targeted monitored parameters compared to standard management.

**Design:**

Prospective cohort study of patients undergoing encephaloduroarteriosynangiosis surgery versus standard neurovascular interventions. Patients with ERSIAS had strict perioperative management that included normocapnia and intentional hypertension. Control patients received regular anaesthetic standard of care. Minute-by-minute intraoperative vitals were electronically collected. Heterogeneity of variance tests were used to compare variance across groups. Mixed-model regression analysis was performed to establish the effects of treatment group on the monitored parameters.

**Setting:**

Tertiary care centre.

**Participants:**

24 participants: 12 cases (53.8 years±16.7 years; 10 females) and 12 controls (51.3 years±15.2 years; 10 females). Adults aged 30–80 years, with transient ischaemic attack or non-disabling stroke (modified Rankin Scale <3) attributed to 70–99% intracranial stenosis of the carotid or middle cerebral artery, were considered for enrolment. Controls were matched according to age, gender and history of neurovascular intervention.

**Main outcome measures:**

Variability of heart rate, mean arterial blood pressure (MAP), systolic blood pressure and end tidal CO_2_ (ETCO2) throughout surgical duration.

**Results:**

There were significant reductions in the intraoperative MAP SD (4.26 vs 10.23 mm Hg; p=0.007) and ETCO2 SD (0.94 vs 1.26 mm Hg; p=0.05) between the ERSIAS and control groups. Median MAP and ETCO2 in the ERSIAS group were higher (98 mm Hg, IQR 23 vs 75 mm Hg, IQR 15; p<0.001, and 38 mm Hg, IQR 4 vs 32 mm Hg, IQR 3; p<0.001, respectively).

**Conclusions:**

The ERSIAS anaesthesia protocol successfully reduced intraoperative fluctuations of MAP and ETCO2. The protocol also achieved normocarbia and the intended hypertension.

**Trial registration number:**

NCT01819597; Pre-results.

**Strengths and limitations of this study**

- An evidence-based and expert-opinion perioperative management protocol was developed to reduce intraoperative physiological variability.
- A prospective, controlled-cohort study with electronically collected intraoperative data allowed for the comparison of intergroup variability, while reducing the bias of traditional manually collected anaesthesia vitals.
- Variability and achievement of haemodynamic goals were assessed with continuous intraoperative physiological parameters.
- Limitations of the study include the intra-group heterogeneity due to the age enrolment range and the use of matched control design. We attempt to minimise the intergroup heterogeneity by matching demographics of the patients and selecting the matched controls exclusively from patients with neurosurgical cerebrovascular interventions.
- Future studies evaluating the impact of variability reduction in clinical outcomes will be necessary to confirm the importance of protocols like the one described here.

## Introduction

Reduction in variability, a central tenet of quality control in many disciplines, has gained growing attention in the medical field.^1^ Unintended variations can lead to a reduction in quality and reliability, which may increase the risk for complications; such variations therefore indicate the need for improved treatment methodologies.^2^ In the surgical setting, intraoperative haemodynamics are a common source of variability.^3–8^ Intraoperative hypotension occurs in up to 99% of surgical patients, with perioperative hypertension affecting 25% of surgical patients.^3^ ^4^ Both intraoperative bradycardia and tachycardia have also been reported as common among surgical patients.^5–7^ Applying quality management principles, such as variability reduction, is essential in improving the quality of surgical care.^8^

Intraoperative haemodynamic variability can result in insufficient cerebral perfusion.^9–11^ Patients with intracranial arterial stenosis (ICAS) are particularly susceptible to variations in cerebral perfusion and are at increased risk for perioperative strokes. In patients with symptomatic cerebrovascular disease and pre-existing ischaemic symptoms, a stroke rate of up to 13% has been reported.^12^

In addition to meticulous surgical technique and use of perioperative antiplatelet therapy, strict intraoperative management is necessary to minimise complications. An anaesthesia protocol composed of strict physiological goals with extensive personnel briefings and checks was developed as part of the ongoing Encephaloduroarteriosynangiosis (EDAS) Revascularisation for Symptomatic Intracranial Arterial Stenosis (ERSIAS) trial. The purpose of this protocol was to minimise fluctuations in physiological variables and achieve parameters conducive to adequate cerebral perfusion in patients with ICAS. In the study presented here, we evaluated the hypothesis that the ERSIAS anaesthesia protocol would be able to reduce the intraoperative variability of patient vitals compared to non-stenosis-related vascular neurosurgical interventions used in a control group. Future studies will be required to evaluate the impact of variability reduction on clinical outcomes.

## Methods

### Study design

To evaluate the impact of the ERSIAS anaesthesia protocol on intraoperative physiological parameters during EDAS surgery, we performed a prospective controlled cohort study of the patients enrolled in the ERSIAS trial (clinicaltrial.gov # NCT01819597) at a tertiary care centre from March 2013 to March 2015.^13^ Adults aged 30–80 years, with transient ischaemic attack or non-disabling stroke (modified Rankin Scale <3) attributed to 70–99% intracranial stenosis of the carotid or middle cerebral artery and confirmed by catheter angiography, were considered for enrolment. All patients had failed intensive medical management and were presenting symptoms attributable to hypoperfusion in the compromised vessel vascular territory, confirmed in perfusion MRI studies. The study was conducted with Institutional Review Board approval (IRB# 12-000439) and participants gave informed consent before taking part.

A matching algorithm was used to identify a control group of individuals from our institutional departmental database with a 1:1 allocation ratio. Controls were identified matching for age within 5 years, gender and a neurovascular intervention, including arteriovenous malformation resection and aneurysm clipping. To minimise selection bias, matches with most recent surgery dates identified by the algorithm were selected as controls.

### EDAS anaesthesia protocol design

A detailed, evidence-based and expert-opinion perioperative management protocol was developed for the ERSIAS trial. The evidence portion was extrapolated from the results of the International Stroke trial and the GESICA (Grupo de Estudio de la Sobrevida en la Insuficiencia Cardiaca en Argentina) study. Then expert neuroanaesthesiologists (BVDW, KZ and AB) worked with the surgical team to define the specific goals. The protocol requires establishment of a systolic blood pressure (SBP) baseline during the preoperative evaluation with measures of SBP in the recumbent, sitting and standing positions to the level at which the patient does not present symptoms suggestive of hypoperfusion. The goal SBP level is set at 20% over the patient's known baseline, unless the patient is hypertensive at baseline (SBP ≥140 mm Hg). For hypertensive patients, the asymptomatic SBP level determined during the preoperative evaluation is selected for the intraoperative goal. End tidal CO_2_ (ETCO2) is kept between 35 and 45 mm Hg, and hyperventilation is avoided. Fluid balance is targeted at euvolaemia to 1.5 L hypervolaemia, with early replacement of the calculated volume deficit due to the nothing by mouth (NPO) time before the surgery. In general, the medications used for all cases are propofol, remifentanil, rocuronium and fentanyl for the induction, sevoflurane, remifentanil and rocuronium for the maintenance, and phenylephrine (as intermittent boluses or infusions) or occasionally norepinephrine or epinephrine for blood pressure support. Table 1A–C provides details for all study participants.

**Table 1:** Anaesthesia management data

Panel (A)										
				Anaesthesia induction and maintenance						
Subject ID	Group	Weight (kg)	Surgery duration (min)	Rocuronium* (mg)	Fentanyl* (µg)	Remifentanil* (µg)	Remifentanil Drip (µg/kg/min)	Propofol* (mg)	Sevoflurane (Y or N)	Desflurane (Y or N)
1	Control	68	321	120	0	772	0.05–0.2	180	Y	N
2	Control	73.9	373	50	0	240	0.05–0.1	230	Y	N
3	Control	83.5	536	140	250	747	0.05–0.1	290	Y	N
4	Control	86.9	429	130	0	240	0.02–0.1	250	Y	N
5	Control	77.9	157	50	500	0	0	0	N	N
6	Control	88.4	519	70	0	352	0.04–0.15	400	Y	N
7	Control	85.3	750	100	0	0	0.02–0.5	240	Y	N
8	Control	72	396	130	125	280	0.05–0.15	200	Y	N
9	Control	67	329	100	0	433	0.02–0.1	490	Y	N
10	Control	87.2	193	80	250	0	0.1–0.15	0	Y	N
11	Control	68.5	408	100	0	0	0.02–0.1	180	Y	N
12	Control	54.4	223	50	0	100	0.04–0.1	460	Y	N
13	EDAS	82	403	140	0	0	0.02–0.1	500	Y	N
14	EDAS	65	543	110	25	0	0.03–0.1	310	Y	N
15	EDAS	95.7	454	100	250	240	0.01–0.25	180	Y	N
16	EDAS	75.4	484	110	225	0	0	250	Y	N
17	EDAS	63.9	522	100	250	0	0	750	Y	Y
18	EDAS	81	446	130	250	0	0	200	Y	N
19	EDAS	63	428	190	0	80	0.01–0.08	250	Y	N
20	EDAS	64.3	389	100	0	0	0.03–0.1	230	Y	N
21	EDAS	60	447	80	150	0	0.02–0.4	140	Y	N
22	EDAS	52.8	454	80	250	105	0.03–0.1	100	Y	Y
23	EDAS	46.7	442	90	0	160	0.02–0.1	370	Y	N
24	EDAS	52	374	80	0	80	0.05–0.1	410	Y	N

Panel (B)												
				Intraoperative blood pressure management								
Subject ID	Group	Weight (kg)	Surgery Duration (min)	Phenylephrine* (µg)	Phenylephrine Drip (µg/kg/min)	Norepinephrine Drip (µg/kg/min)	Epinephrine* (µg)	Epinephrine Drip (µg/kg/min)	Nitroglycerine* (µg)	Ephedrine* (mg)	Esmolol* (mg)	Labetalol* (mg)
1	Control	68	321	100	0.01–0.4	0	0	0	0	15	0	75
2	Control	73.9	373	250	0	0	0	0	0	20	0	0
3	Control	83.5	536	200	0.05–0.35	0	0	0	0	0	30	10
4	Control	86.9	429	120	0.02–0.15	0	0	0	200	0	0	10
5	Control	77.9	157	0	0	0.1–0.15	0	0	0	0	0	0
6	Control	88.4	519	0	0	0	0	0	0	0	0	20
7	Control	85.3	750	600	0.2–0.5	0	0	0	0	0	0	0
8	Control	72	396	0	0	0	0	0	0	0	0	0
9	Control	67	329	420	0	0	0	0	500	0	0	35
10	Control	87.2	193	0	0	0.02–0.04	0	0	0	0	0	0
11	Control	68.5	408	500	0.2–1	0	0	0	250	0	0	0
12	Control	54.4	223	100	0	0	0	0	0	0	0	0
13	EDAS	82	403	2300	0.2–1	0.05–0.15	5	0	0	25	0	0
14	EDAS	65	543	1975	0.2–1	0	0	0	0	20	0	0
15	EDAS	95.7	454	2730	0.2–1	0	0	0	250	0	100	0
16	EDAS	75.4	484	1950	0.2–0.4	0	0	0	300	20	0	0
17	EDAS	63.9	522	2370	0.05–1	0	0	0	50	0	0	0
18	EDAS	81	446	1200	0	0	0	0	0	85	0	0
19	EDAS	63	428	1900	0.1–0.28	0	45	0	0	45	0	0
20	EDAS	64.3	389	600	0.2–0.5	0	0	0.01–0.13	0	0	0	0
21	EDAS	60	447	550	0.3–1.4	0	0	0	500	55	0	0
22	EDAS	52.8	454	200	0.2–1.2	0	0	0	0	0	0	0
23	EDAS	46.7	442	600	0.3–1	0	0	0	0	5	0	0
24	EDAS	52	374	1125	0.2–1.2	0	0	0	200	5	20	0

Panel (C)									
				Intraoperative fluid and ventilatory management					
Subject ID	Group	Weight (kg)	Surgery Duration (min)	Mannitol (g)	Mean Tidal Volume (mL)	Mean Respiratory Rate (breaths/min)	Normal Saline (mL)	Plasmalyte (mL)	Albumin 5% (mL)
1	Control	68	321	70	355.1	13.6	1000	1800	0
2	Control	73.9	373	37	529.4	11.3	1000	1000	0
3	Control	83.5	536	80	502.9	12.7	1000	2700	0
4	Control	86.9	429	63	534.3	11.1	1000	2500	0
5	Control	77.9	157	0	498.5	11.3	500	500	0
6	Control	88.4	519	20	415	13.1	0	3000	0
7	Control	85.3	750	67	500.7	11	0	5100	0
8	Control	72	396	70	449	13.9	1000	2600	0
9	Control	67	329	67	478.3	11.1	1000	3700	0
10	Control	87.2	193	100	471.1	17.8	51	1400	0
11	Control	68.5	408	68	464.1	12.5	1000	2500	0
12	Control	54.4	223	100	404.2	11.6	4000	1000	0
13	EDAS	82	403	0	530.6	12.1	0	5100	0
14	EDAS	65	543	0	481.3	8.9	1274	3800	250
15	EDAS	95.7	454	0	444.8	9.23	600	1800	0
16	EDAS	75.4	484	0	421.3	11.3	1500	2300	0
17	EDAS	63.9	522	0	455.2	8.84	1000	2900	0
18	EDAS	81	446	0	588.6	12.6	1000	3400	500
19	EDAS	63	428	0	446.7	13.2	500	2300	350
20	EDAS	64.3	389	0	490.6	14.2	1000	3300	0
21	EDAS	60	447	0	426.8	11.9	0	3000	0
22	EDAS	52.8	454	0	313.8	13.8	1000	3000	0
23	EDAS	46.7	442	0	343.6	11.3	1000	6500	0
24	EDAS	52	374	0	486.54	8	1500	2750	250

*Total administered incrementally during the case.

EDAS, encephaloduroarteriosynangiosis.

Vitals are monitored with standard ECG monitoring, an arterial line and central venous pressure monitoring. Aspirin (325 mg) is administered on the date of surgery and for at least 3 days prior. Body temperature is maintained between 35.5 and 36.5°C. Barbiturates, steroids (except dexamethasone for nausea) and mannitol are not administered during the procedure.

All involved parties are alerted via electronic mail and pager of upcoming EDAS cases. On the day of the operation, the surgical, anaesthesia, electrophysiology and nursing teams review, line by line, an ERSIAS Surgical-Anesthesia Management Briefing with a detailed checklist. In this process, there is confirmation of completion of basic presurgical safety practices from all team members—surgeons, anaesthesiologists, nurses and technicians—prior to the administration of anaesthesia. The patient-specific goals for anaesthesia management are confirmed during the briefing, and audible alarms are set to indicate deviations from intended goals. Monitors are strategically positioned to allow the entire surgical staff continuous visualisation of all patient physiological parameters. The ERSIAS Surgical-Anesthesia Management Checklist is included as an online supplementary list.

### Patient involvement

Patients, service users, carers and laypeople were not involved in setting the research question, the outcome measures, the design of the study or the dissemination of its results.

### Data collection

Minute-by-minute physiological parameters were electronically collected throughout the duration of surgery using the electronic medical record system CareConnect (Epic Systems, Madison, Wisconsin, USA). The recorded physiological parameters include heart rate (HR), respiration rate, O_2_ saturation, temperature, ETCO2, central venous pressure, SBP, diastolic blood pressure, MAP, central venous pressure, urine output and temperature. Surgical duration was defined as the period between patient arrival and departure from the operating room.

### End point measures

The end point measure of this study is the variability of HR, MAP, SBP and ETCO2 throughout the surgical duration.

### Statistics

Statistical analysis was performed with JMP (V.11, SAS Institute, Inc., Cary, North Carolina, USA, 1989–2007). Descriptive statistics were prepared with the use of contingency table analyses for categorical data and Fisher's exact test. Student's t test was used to compare continuous, normally distributed data. The Wilcoxon rank-sum test was used to compare continuous, non-normal data. Power calculations were performed using Bartlett's test, which follows the χ^2^ distribution. On the basis of this distribution with an α error probability of 0.05, a total sample size N=20 was estimated to detect a variance ratio difference of 0.37 with a power of 0.81 Heterogeneity of variance tests were performed to compare variances across groups using an analysis of means for variances based method. This method indicates whether any of the group SDs are different from the square root of the average variance. To be robust against non-normal data, the method uses a permutation simulation to compute decision limits. The permutation simulation analyses the distribution of model outputs as a function of the random variation in the factors and the model noise. The complete details of the method can be found in Wludyka and Sa.^14^ Groups exceeding the computed decision limits were concluded to have variances statistically different from the square root of the average group variance. To further confirm the heterogeneity of variance, a Bartlett test was performed to test that the variances were equal. One-way ANOVA was used when two groups had equal variances. To evaluate the effects of the protocol on the specific targeted monitored parameters, a mixed-model regression for repeated measures was performed. The first-order autoregressive (AR[1]) covariance structure was used, random effects were assigned to account for intersubject variability, and the measurements were nested by subject. This provides an adequate correlation structure for repeated measures in time.

## Results

**Figure 1:**
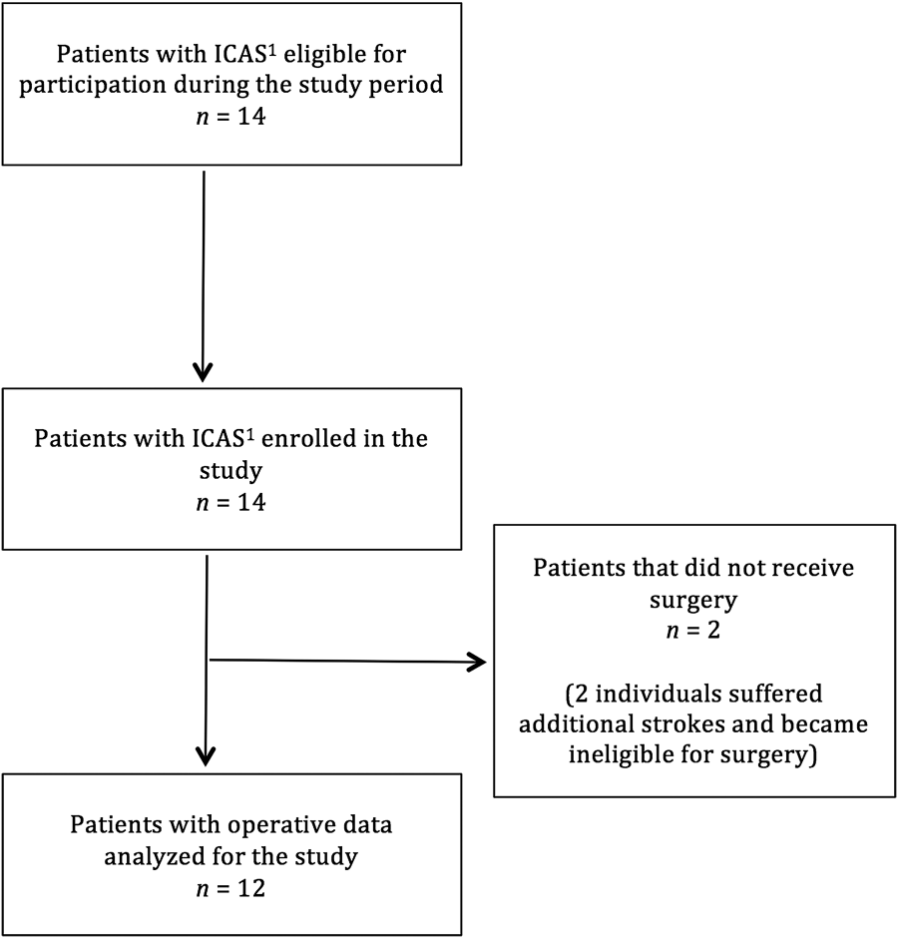
Flow diagram of patients examined for eligibility and included in the ERSIAS (Encephaloduroarteriosynangiosis Revascularization for Symptomatic Intracranial Arterial Stenosis) group. ICAS, intracranial arterial stenosis.

**Figure 2:**
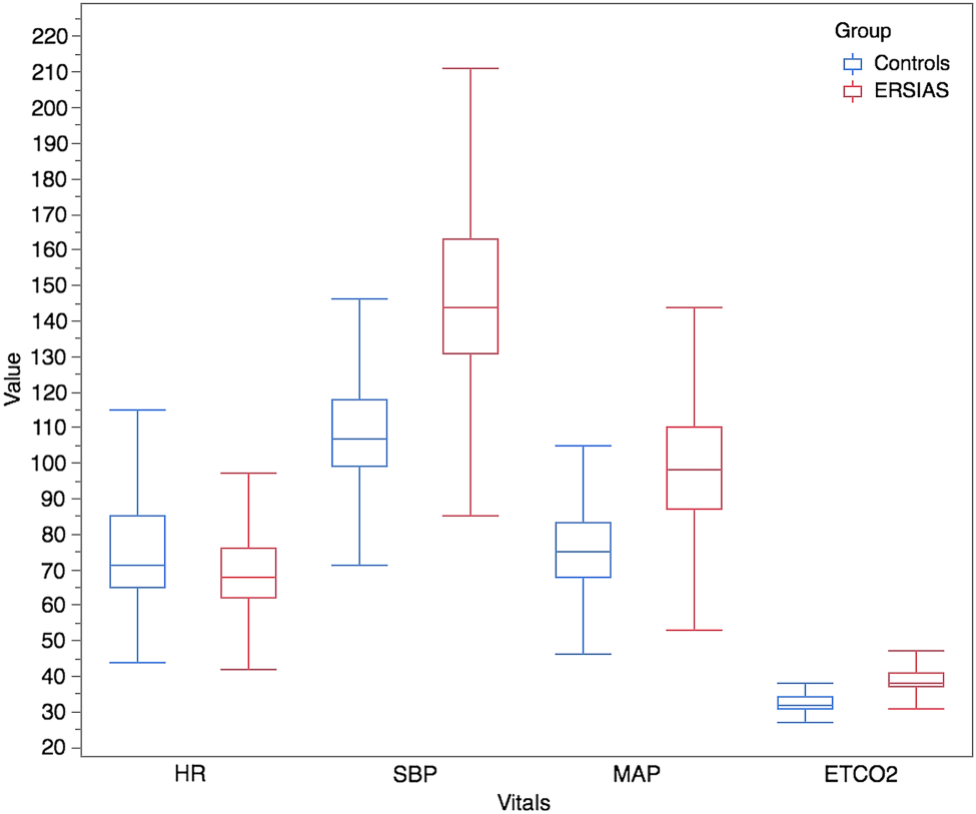
Box plot of end point vitals per group. Distribution of 3 219 917 data points obtained by minute-to-minute electronic data collection in 24 patients (12 ERSIAS and 12 matched controls). HR, heart rate; ETCO2, end tidal CO_2;_ MAP, mean arterial blood pressure; SBP, systolic blood pressure.

Of the 14 patients enrolled to undergo EDAS, 2 suffered additional strokes before any surgery was performed and became ineligible for the operation (figure 1). This group of 12 patients is the ERSIAS group, which consists of 10 females and 2 males, with a mean age of 53.8 years (SD=16.7 years). Twelve control patients (10 females, 2 males) were identified using the matching algorithm with a mean age of 51.3 years (SD=15.2 years). Ten (83.3%) underwent aneurysm clipping, and 2 (16.6%) had an arteriovenous malformation resection. The total surgical duration of the EDAS group was 5386.0 min (SD=49.8 min), and that of the control group was 4634.0 min (SD=164.9 min). There were no significant differences in the mean age or surgical duration between groups. Demographics are summarised in table 2. No major adverse events were observed throughout this study, and all patients completed their surgical intervention. In particular, no patient suffered either intraoperative or immediate postoperative ischaemic or haemorrhagic strokes in the ERSIAS group. Figure 2 represents the distribution of the end point data—HR, SBP, MAP and ETCO2. Patient physiological parameters are summarised in table 3.

**Table 2:** Summary of demographics and total surgery duration between ERSIAS (Encephaloduroarteriosynangiosis Revascularization for Symptomatic Intracranial Arterial Stenosis) and control groups

Demographics	Control group	ERSIAS group
Number of patients	12	12
Mean age±	51.3 years±15.2	53.8 years±16.7
Gender		
Female	10 (83.3%)	10 (83.3%)
Male	2 (16.6%)	2 (16.6%)
Procedure		
EDAS	0	12 (100%)
Aneurysm	10 (83.3%)	0
AVM	2 (16.6%)	0
Surgery duration (min)		
Mean	386.2	448.8
SD	157.9	47.7
Median	384.5	446.5
IQR	249.0	67.3

AVM, arteriovenous malformation; EDAS, encephaloduroarteriosynangiosis.

**Table 3:** Summary of Iintraoperative Pphysiological Pparameters between ERSIAS (Encephaloduroarteriosynangiosis Revascularization for Symptomatic Intracranial Arterial Stenosis) and control groups

	Control group	ERSIAS group	
Vitals	Median IQR	Median IQR	p Value
Heart rate (bpm)	71 20	68 14	0.005
Systolic blood pressure (mm Hg)	107 19	144 32	0.001
Mean arterial blood pressure (mm Hg)	75 15	98 23	0.001
End tidal CO_2_ (mm Hg)	32 3	38 4	0.001

Median and IQR are presented, as variables were not of normal distribution. To account for repeated measures, a mixed model regression was used. The p values reported correspond to the parameter estimates of each variable.

There were significant reductions in the intraoperative variability of MAP and ETCO2 in the ERSIAS group below the lower-boundary decision limit computed by using a permutation simulation. The variability of the HR and SBP was not significantly different between the ERSIAS and control groups during the surgical period. There were significant reductions in the intraoperative MAP SD and ETCO2 SD between the ERSIAS and control groups. The MAP SD of the ERSIAS group was 4.26 mm Hg, while that of the control group was 10.23 mm Hg (p=0.007). The ETCO2 SD for the ERSIAS and control groups was 0.94 and 1.26 mm Hg, respectively (p=0.05). There were no significant differences in the intraoperative HR SD and SBP SD between groups.

As intended by protocol design, the intraoperative median MAP and ETCO2 in the ERSIAS group were higher than in the control group. The intraoperative median MAP was 98 mm Hg (IQR 23) for the ERSIAS group and 75 mm Hg (IQR 15) for the control group, p<0.001. The intraoperative median ETCO2 was 38 mm Hg (IQR 4) for the ERSIAS group and 32 mm Hg (IQR 3) for the control group, p<0.001. The median HR of the ERSIAS group (68 bpm, IQR 14) was lower than that of the control group (71 bpm, IQR 20; p=0.005) over the surgical duration. The intraoperative median SBP of the ERSIAS group was 144 mm Hg (IQR 32), while that of the control group was 107 mm Hg (IQR 19), p<0.001.

## Discussion

Consistent and reliable application of quality improvement principles to healthcare has significant positive effects on patient outcomes.^15^ ^16^ Among the most relevant principles applicable to surgical specialties is the minimisation of unintended variability, which translates into error reduction and increased consistency in procedural results.^8^ While every case requires specific goals, detailed, comprehensive protocols and practices for procedural standardisation and reproducibility are fundamental to the future of evidence-based medicine. The goal of this study was to evaluate the effectiveness of the ERSIAS anaesthesia protocol in minimising intraoperative haemodynamic variation compared to standard neurovascular interventions. The importance of this goal was to achieve the intended benefit for the enrolled patients, as well as to serve as a model for the future evaluation of procedures for patients with or at risk of stroke. The benefit in terms of standard outcomes, including stroke and mortality, has not as yet been demonstrated, and will require further investigation. Our study demonstrated a reduction in the variability of MAP and ETCO2 during the surgical period. This study also demonstrated successful and consistent increases in intraoperative median MAP and ETCO2 in the ERSIAS group, as intended by the protocol, given the specific needs of the patients treated. By using electronically collected data in this study, we were able to avoid the bias that is introduced by the traditional manual collection of anaesthesia vitals. This method represents a valuable application of the large body of information that current electronic record management can provide.

The reduction of unintended variability around targets can have important effects following surgery. There is increasing evidence that perioperative haemodynamic variability has a negative impact on postsurgical clinical outcomes.^11^ ^17–19^

In a recent large cohort of patients who underwent major non-cardiac surgery, intraoperative variance in blood pressure was found to be significantly associated with postoperative delirium.^20^ In a study of cardiac surgery patients, intraoperative SBP variability was determined to be associated with increased 30-day postoperative mortality, proportional to the degree of SBP excursion from a specific intraoperative range.^21^ ^22^ Increased perioperative blood pressure variability was also associated with increased time to extubation and hospital stay.^11^ Perioperative blood pressure variability has also been reported to increase the risk of stroke, myocardial ischaemia and bleeding.^11^ ^18^ Methodologies to reduce variability may contribute significantly to the improvement of surgical care.

The inclusion of preoperative preparation and alerts, intraoperative detail briefing with the participation of all the involved personnel, and the creation of and adherence to a checklist in the ERSIAS protocol were practical measures that had an objective impact in reduced variability. Checklists have been shown to increase adherence to care processes, to increase standardisation, and to reduce errors.^23–25^ In a study on the WHO Surgical Safety Checklist conducted on non-cardiac surgical patients in eight hospitals worldwide, inpatient complications were reduced from 11% to 7%, and mortality decreased from 1.5% to 0.8%.^26^ Supplementing a checklist with structured briefings among team members also contributes to a reduction in complications and mortality.^27^ ^28^ These processes have also enhanced communication and situational awareness among team members, which ultimately improves surgical outcomes.^25^ ^27^

Adequate cerebral perfusion pressure in patients with ICAS is necessary to sufficiently perfuse cerebral tissue.^29^ The ERSIAS anaesthesia protocol was developed to meet specific physiological targets. There is a lack of specific literature in regard to the effects of blood pressure management in the perioperative period of patients with intracranial stenosis of atherosclerotic origin. It is well known that, for those individuals responding to medical management, strict prevention of hypertension plays an important role in reducing their risk of stroke; 30 however, for those patients failing medical management, in which the aetiology of the stroke is not artery-to-artery embolism but hypoperfusion, often the only measures available to manage their symptoms are volume expansion and moderate hypertension. In GESICA,^31^ patients were considered to have clinically significant stenosis if they developed symptoms during changes of position, effort, or during the introduction or increase in dose of an antihypertensive drug. This group of patients had a subsequent rate of combined stroke and transient ischaemic attack (TIA) of 61%. In cases of acute stroke (of all aetiologies), the International Stroke trial investigators showed a ‘U shaped’ relationship between baseline SBP and primary outcomes of death within 14 days and death or dependency at 6 months.^32^ The lowest frequency of poor outcome was found between 140 and 179 mm Hg.^32^ The rationale in the ERSIAS trial to aim for an SBP goal relatively ‘hypertensive’ compared with normal participants is based on those observations. For optimal perfusion across stenotic vessels and collaterals, blood pressure was maintained to patient-specific needs (baseline asymptomatic or 20% over the preoperative blood pressure baseline).^33^ ^34^ This was supplemented by ensuring normovolaemia to a slightly hypervolaemic fluid state, early during the operation. To avoid cerebral vasoconstriction, intraoperative ventilation was targeted to normocapnia, while avoiding hyperventilation.^29^ ^34^ To reduce the stroke risk associated with embolic events in ICAS, perioperative, full aspirin doses were maintained.^29^ ^34^

The ERSIAS anaesthesia protocol examined in this study produced a reduction in physiological parameter variability, promoting consistent conditions favourable to cerebral perfusion. Beyond improving the quality of surgical care for patients at risk of stroke, attention to standardised anaesthesia and perioperative protocols is key to reduce confounders in the evaluation of surgical or interventional techniques in clinical trials. Recent trials (the Stenting vs Aggressive Medical Therapy for Intracranial Arterial Stenosis (SAMMPRIS) trial, the Carotid Occlusion Surgery Study (COSS) and the Vitesse Intracranial Stent Study for Ischemic Therapy (VISSIT)) have failed to prove the benefit of certain interventions in stroke management.^35–40^ Several authors have suggested that general anaesthesia may negatively affect endovascular interventions for stroke.^41–45^ However, little attention has been given to the standardisation and adherence to protocols directed at avoiding unfavourable haemodynamic conditions that can significantly affect patients at risk of or with strokes. In several of these trials, the majority of adverse events have occurred during the immediate operative or postoperative period. In SAMMPRIS, 25 of 33 events occurred within 24 h of the percutaneous transluminal angioplasty and stenting procedure.^36^ ^44^ In COSS, 12 out of 14 ipsilateral hemispheric strokes occurred within 48 h of the surgical bypass procedure.^39^ In the cohort we are presenting, no patients suffered ischaemic or haemorrhagic strokes during the surgery or perioperative period. Although this result cannot only be attributed to anaesthetic management, the outcomes are better when compared with prior reports of similar patients with IAS. Komotar *et al*^46^ reported a perioperative rate of stroke of 33% for patients who underwent EDAS for IAS. Although the effectiveness of the techniques being investigated most likely played a key role in the results, additional aspects beyond the procedure and related to ensuring adequate cerebral perfusion should be controlled in the future.

## Conclusions

The ERSIAS anaesthesia protocol was effective in reducing variability of intraoperative physiological parameters and achieving the haemodynamic goals established for patients with ICAS undergoing EDAS surgery. The application of protocol and standard practices to reduce intraoperative variability may prove to be an important addition to future large-scale clinical trial protocols attempting to evaluate the efficacy of a treatment or surgical technique, minimising the confounding effect of variations in anaesthetic management on patient outcomes.

Data sharing: The relevant anonymised patient level data are available on reasonable request from the corresponding author based on the following criterion: scientific merit of individual placing request, sufficient power within the data to address the aims of the request, likelihood that the effort will be completed through publication, resources necessary to support the required analysis/publication, appropriate approval obtained from the Institutional Review Board, if necessary. If data request is approved, data and the associated documentation will be made available to users only under a Data Use Agreement that provides for: (1) a commitment to using the data only for research purposes, (2) a commitment to securing the data using appropriate computer technology, and (3) a commitment to destroying or returning the data after analyses are completed (UCLA Policy HS 9440).

Identifiable patient data: The manuscript does not contain personal medical information about an identifiable living individual.
